# Differences in Brain Function and Changes with Intervention in Children with Poor Spelling and Reading Abilities

**DOI:** 10.1371/journal.pone.0038201

**Published:** 2012-05-31

**Authors:** Daniela Gebauer, Andreas Fink, Reinhard Kargl, Gernot Reishofer, Karl Koschutnig, Christian Purgstaller, Franz Fazekas, Christian Enzinger

**Affiliations:** 1 Department of Neurology, Medical University of Graz, Graz, Austria; 2 Department of Psychology, Karl-Franzens-University Graz, Graz, Austria; 3 Institute for Reading and Spelling, Graz, Austria; 4 Department of Radiology, Medical University of Graz, Graz, Austria; Yale University, United States of America

## Abstract

Previous fMRI studies in English-speaking samples suggested that specific interventions may alter brain function in language-relevant networks in children with reading and spelling difficulties, but this research strongly focused on reading impaired individuals. Only few studies so far investigated characteristics of brain activation associated with poor spelling ability and whether a specific spelling intervention may also be associated with distinct changes in brain activity patterns. We here investigated such effects of a morpheme-based spelling intervention on brain function in 20 children with comparatively poor spelling and reading abilities using repeated fMRI. Relative to 10 matched controls, children with comparatively poor spelling and reading abilities showed increased activation in frontal medial and right hemispheric regions and decreased activation in left occipito-temporal regions prior to the intervention, during processing of a lexical decision task. After five weeks of intervention, spelling and reading comprehension significantly improved in the training group, along with increased activation in the left temporal, parahippocampal and hippocampal regions. Conversely, the waiting group showed increases in right posterior regions. Our findings could indicate an increased left temporal activation associated with the recollection of the new learnt morpheme-based strategy related to successful training.

## Introduction

According to the ICD 10 definition [1], spelling and reading impairment (dyslexia; F 81.0) is diagnosed if reading and spelling skills are located two standard deviations below the level that might be expected based on general intelligence, age and education. The main feature of isolated spelling disorder (F 81.1) is a specific and significant impairment in the development of spelling skills in the absence of a history of specific reading disorder, which is not accounted for by age, intelligence or inadequate education. However, it has to be noted that difficulties in spelling and reading occur in various degrees of severity. Although cut points are placed to help define groups, they have been criticized for being arbitrary and lack biological validity [2]. Reading difficulties, including dyslexia, occur as the lower part of a continuum for reading ability that includes nonimpaired as well as disabled readers [2], [3]. In this study we thus conceptualize spelling and reading ability as a continuum ranging from lower to higher abilities, rather than relying on categorical definitions such as “dyslexic” or “spelling impaired” children. In defining our experimental groups, we chose children exhibiting a comparatively poor performance level on this ability continuum. Discoveries about poor spelling and reading abilities may offer insights into mechanisms of normal reading acquisition and dyslexia [3].

Recent studies using functional magnetic resonance imaging (fMRI) provided important insights into potential brain mechanisms underlying reading and spelling skills and reading and spelling impairment (i.e., dyslexia; for a recent fMRI study on developmental dyslexia in the German-speaking area see e.g. [4]). Frequently, *decreased* brain activation in parieto-temporal and occipito-temporal regions of the *left* hemisphere, along with *increased* activation in frontal and right hemispheric language-related regions has been observed in individuals with reading and spelling impairment. The left parieto-temporal region (angular gyrus and supramarginal gyrus) is assumed to play a critical role in spelling in non-impaired individuals [5], [6]. Decreased left parieto-temporal activation has been related to deficits in grapheme-phoneme-conversion in reading and spelling impairment [7]. The left occipito-temporal region (comprising the visual word form area) is crucial for reading processes in non-impaired individuals [8]. Decreased activation in this region is associated with impairments of automatic, fluent reading [7], [9]. Also increased frontal and right hemispheric activation has been observed in individuals with reading and spelling impairment, interpreted to indicate inefficient compensatory mechanisms such as internal articulation [10], [11].

Training studies using fMRI revealed changes in the above mentioned brain activation patterns in subjects with reading and spelling impairment and poor readers along with successful intervention [12]–[20], while only few studies focused on subjects with poor spelling ability [21].

Isolated spelling difficulties in German-speaking samples were found in about 3–6% of elementary school children [22], [23]. Due to the transparent orthography in German, individuals with reading and spelling impairment manage to read slow but accurate in the course of their development, whereas spelling mistakes rather persist into adulthood [24].

Here, we used a morpheme-based spelling intervention (*Morpheus*) [25], which trains children to figure out the correct spelling of a word by separating it into specific word component parts (*morphemes*). Behavioral studies in this field provided evidence that such interventions significantly enhance reading and/or spelling ability [26]–[29]. Using EEG, we demonstrated a neurophysiological training effect of this intervention, by increased EEG activation in left hemispheric regions that are involved in the complex neural network subserving reading and spelling [29].

Using fMRI in a different sample, we sought to corroborate and extend these findings by more detailed functional neuroanatomical insights. We specifically tested (a) if and how brain activation patterns at baseline in children with comparatively poor spelling and reading abilities differed from controls. We hypothesized that children with poor spelling and reading abilities would show different brain activation prior to the applied spelling intervention as compared to controls. Secondly (b), we investigated whether spelling skills and brain activation can be modulated by a specific spelling intervention, comparing two groups of poor spellers (divided into a training group and into a waiting group).

## Materials and Methods

### Ethics Statement

The study was approved by the ethics committee of the Medical University of Graz, Austria. All children and their parents had given written informed consent.

### Psychometric Tests

In an extensive pre-experimental screening, standardized tests for the assessment of reading and spelling abilities were administered in 107 children, and we explored relevant socio-demographic data such as age, sex and native language.

To assess spelling skills, we used a standardized spelling test (Hamburger-Schreibprobe, HSP) by May et al. [30]. In the HSP, words and sentences are dictated by the experimenter and have to be written next to the corresponding pictures that illustrate the respective words or sentences. This test takes about 15 minutes. Within this study, versions for 4^th^/5^th^ graders and 5^th^ to 9^th^ graders were applied. The HSP provides measures for the number of correctly spelled words and the number of grapheme-related mistakes. The latter measure was used in this study as it provides a more precise measure of spelling ability.

Additionally, we administered the “Salzburger-Lese-Sreening” (SLS) [31], [32] that measures reading speed and basic reading ability (automaticity). The SLS 1–4 was used for children up to the 4^th^ grade, and the SLS 5–8 was applied for older children and parallel versions exist for both. In the SLS, children have to decide whether the content of a presented sentence is correct or not. Testing time is limited to three minutes. In addition, we also measured reading comprehension (i.e. comprehension of words, sentences and text) by means of a standardized German-speaking test (ELFE 1–6) [33]. Furthermore, non-verbal intelligence was measured by the Standard Progressive Matrices (SPM) by Raven [34].

### Participants

Forty-two German-speaking children aged between nine and 15 years were recruited for this study based on the pre-experimental screening as described above (cf. 2.2). Three groups (training group, waiting group and control group), each comprising 14 children, were investigated. Children with overall motion >3 mm or sudden movement >1 mm during scanning were excluded from further analyses. Based on this definition, seven children had to be excluded due to movement artifacts. Furthermore, two children had to be excluded due to poor behavioral performance inside the scanner (Mean Accuracy <70%) and three children had to be excluded because they did not attend all behavioral tests and fMRI sessions, rendering a final sample of 30 children (15 males), whose age ranged from 10 to 15 years (*M* = 11.80; *SD* = 1.58, see Table 1). All participants were right-handed, healthy and had normal or corrected-to normal vision.

**Table 1:** Descriptive Statistics of Behavioral Measures (sex, age, non-verbal intelligence, reading- and spelling skills).

	TG	WG	CG	p
**Behavioral Measures**				
Sex	10 (7 males)	10 (5 males)	10 (3 males)	
Age (years)	11.5 (+/−0.7)	11.6 (+/−1.7)	12.3 (+/−2.1)	.49
Intelligence – Raven raw scores	36.7 (+/−7.7)	36.5 (+/−9.16)	43.4 (+/−5.4)	.09
**Pre-Intervention**				
Reading Skills –sls *	91.4 (+/−14.3)	97.7 (+/−10.4)	115.3 (+/−15.1)	**.001**
Reading Comprehension	48.3 (+/−8.8)	50.9 (+/−5.9)	62.3 (+/−7.9)	**.001**
Spelling Skills -hsp	21.0 (+/−11.4)	23.2 (+/−14.0)	75.7 (+/−14.7)	**.000**
**Post- Intervention**				
Reading Skills -sls	102.6 (+/−13.9)	100.0 (+/−9.1)	-	.53
Reading Comprehension	52.6 (+/−8.7)	50.5 (+/−5.4)	-	.62
Spelling Skills -hsp	42.3 (+/−23.0)	23.9 (+/−13.3)	-	**.04**

Means and Standard Deviations (in brackets).

Pre-Intervention: Reading Skills: SLS Reading Quotient: Average Scores range from 85–115 (*F*_(2,27)_ = 8.52; *p*<.001; *η_p_^2^* = .39); Reading Comprehension: ELFE T-scores: (*F*_(2,27)_ = 9.57; *p*<.001; *η_p_^2^* = .42); Spelling Skills: HSP Percent Rank: (*F*_(2,27)_ = 53.26; *p*<.001; *η_p_^2^* = .80).

Post-Intervention: Reading Skills: SLS Reading Quotient (*F*_(1,18)_ = 0.25; *p* = .62; *η_p_^2^* = .01); Reading Comprehension: ELFE T-scores (*F*_(1,18)_ = 0.42; *p* = .53; *η_p_^2^* = .02); Spelling Skills: HSP Percent Range (*F*_(1,18)_ = 4.83; *p*<.05; *η_p_^2^* = .21).

* Three out of the 20 comparatively poor spellers and readers showed below average reading scores (Reading Quotient: 83,75, 73).

We formed and investigated three experimental groups: (1) Ten children with poor spelling abilities (*M* = 21.0 percent rank) were assigned to the “training group” (TG), (2) another ten children with poor spelling abilities (*M* = 23.2 percent rank) were assigned to the so-called “waiting group” (WG, receiving the training after the post-test) and (3) a control group (CG) of ten children (matched for age and intelligence) with good spelling abilities (*M* = 75.7 percent rank), were investigated. The effect of the intervention was examined in a pre-test/post-test design, comparing the two groups of poor spellers (TG and WG). The groups did not differ significantly (*p*>.05) with respect to age and non-verbal intelligence, but controls scored considerably higher in reading and spelling. Specific post-hoc comparisons by means of the Tukey HSD test revealed that controls showed significantly higher scores of reading and spelling ability than both groups of poor spellers (*p*<.05). Therefore we labeled the groups as “comparatively poor spellers and readers”. However, it has to be noted that the TG and WG showed average reading scores according to age- and education-matched norms (*p*<.01; see Table 1 for details). The comparisons between the TG and the WG yielded no significant results (which appears to be particularly important in the light of the employed training design).

### Intervention

The applied intervention is a computer-aided morpheme-based spelling training (*Morpheus*) [30], which has been approved as an evidence-based intervention for individuals with reading and spelling deficits by the federal ministry of Austria [35] and has shown to significantly improve spelling ability in children in a series of behavioral studies in our laboratory [36], [37].

A morpheme is defined as the “smallest meaningful unit of language” [38]. Every word is built by different parts, which follow particular spellings (e.g. unforgetful = prefix (un), suffix (ful), root (forget)). Therefore, the spelling of the German verb “verfahren” can be derived by two rules: the prefix (ver) is always written with (v), the root (fahr) always with an “h”. Children do not need to remember the spelling of every single word, but only to memorize the spelling of their component parts. Furthermore, morphosemantic information can support the development of a meaning-oriented decoding strategy, e.g. the correct spelling of the noun “Motor-rad” (motor-bike) can be derived by the meaning [30]. In addition, this strategy seems to be easy to apply as only “100 of the most frequent morphemes cover 70% of all written material” [39].

The *Morpheus*-intervention consists of computerized tutorials, a book of exercises and morpheme-based games to facilitate the consolidation of the strategy. The intervention, which includes daily handwritten and computer homework along with instructor-guided courses (once a week, lasting approximately two hours), was realized within *five weeks*. These tutorials on the computer include twelve different playful exercises dealing with morphemes (e.g. recognizing and matching word families, morphological clozes, finding suffixes and prefixes). During the tutorials achieved scores are displayed on the computer screen. Participants can only reach the next difficulty level of the same exercise when they have solved at least 75% of the given problems correctly. The automatically saved score of every tutorial serves as basis for assessing training progress.

The training material of *Morpheus* consists of the most frequent morphemes of the German language and contains different levels of difficulty. The words used for the training were taken from an empirically-based collection of words (German basic vocabulary for 4^th^ graders) [40]. *Morpheus* has been constructed on the basis of the following principles: simplicity, relief due to morpheme segmentation, rule-governed repetition, playfulness, avoidance of mistakes, individuality, productivity, and practicing handwriting.

### Functional MRI (fMRI) experimental stimuli and tasks

Three different lexical decision conditions were presented during event-related fMRI (1: correctly spelled words, 2: misspelled words, 3: pseudowords). Similarly to the spelling judgment task of Richards et al. [21], children had to decide whether a presented word was spelled correctly (e.g. Bäume; trees) or if it was spelled incorrectly (e.g. Menner instead of Männer; men) or a pseudoword (e.g. Ostablast). Misspelled and Pseudowords were created by changing correctly spelled words (nouns, verbs, adjectives). The misspelled words are phonologically correct (sound exactly like the properly spelled word if spoke aloud), like the pseudohomophones [41], [42], but morphematically incorrect, following the “known” pattern of german words (consonant-vocal-consonant-vocal-consonant; e.g. Negel instead of Nägel (nails); forher instead of vorher (previously)). Pseudowords included phonologically non-existing and morphematically incorrect words. A fixation cross was presented as a baseline. We found substantial correlations between spelling ability (assessed by the HSP) and performance during lexical decision tasks (*r* = about .50) in prior studies [29]. Participants were familiarized with the task outside the scanner to ensure the instruction had been understood properly. Each condition comprised 75 words which were equal according to length and word type (25 nouns, 25 verbs, 25 adjectives; mean word length: 7 letters).

**Figure 1:**
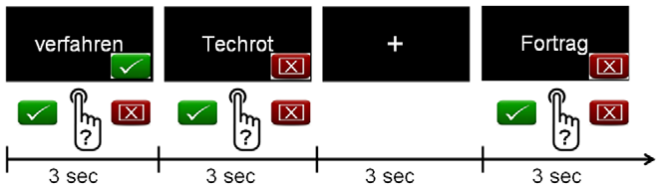
fMRI Paradigm. Correctly spelled words, misspelled words, pseudowords and a fixation cross were presented in a randomized order for three seconds. In each lexical decision condition, children were instructed to respond by either pressing the “correct” button with the index finger or the “misspelled/pseudoword” button with the middle finger on the response console. Responses were given with the right hand and recorded and logged for further analyses. The children did not receive feedback to their responses.

Answers were given via button presses using the right (dominant) hand, with the index finger for correctly spelled real words and the middle finger for misspelled and pseudowords (see Figure 1). Behavioral responses inside the scanner were assessed to obtain the percentage of correct responses and reaction time. Items and fixation were presented for three seconds. Each condition was directly followed by the other. The order of items and fixations was optimized by a genetic algorithm for hemodynamic response detection [43]. The total time of the fMRI experiment was 16 minutes and the entire MRI session took 30 minutes.

### Magnetic Resonance Imaging (MRI) data acquisition and analysis

Imaging was performed on a 3.0 Tesla Trio Tim scanner (Siemens Medical Systems, Erlangen, Germany) using a 12-channel head coil. To minimize head movement, childrens' heads were stabilized with foam cushions. A high-resolution isotropic (1×1×1 mm) structural scan (TR = 1900 ms, TE = 2.2 ms) was acquired to allow precise registration of functional data to individual anatomy. Structural brain scans were reviewed by an expert and did not show morphological abnormalities. Functional images were acquired using a single-shot gradient echo EPI sequence (TR = 2190 ms, TE = 30 ms, matrix 64×64 mm, FOV = 192, Flip Angle 90°, 36 three mm thick slices). Visual stimuli were synchronized with the MR-scanner using “Presentation” (Neurobehavioral Systems, Albany, CA) and back-projected onto a translucent screen installed on the rear of the scanner bore. Participants watched the screen through a mirror attached on the top of the head coil. Answers were given via a button response box as described above.

Functional MRI data analysis was performed using FEAT (fMRI Expert Analysis Tool; Version 4.1.5., part of FMRIB's Software Library, www.fmrib.ox.ac.uk/fsl). The following preprocessing steps were applied: motion correction using MCFLIRT; non-brain removal using BET; interleaved slice time correction; spatial smoothing using a Gaussian kernel of 6 mm FWHM; and high-pass temporal filtering. Time series statistical analysis was carried out using FILM. Motion parameters were included in the model as covariates of no interest. Nonlinear registration to high-resolution and standard images (Montreal Neurological Institute (MNI) space) was carried out using FNIRT. Higher level analysis was done using FLAME (FMRIB's Local Analysis of Mixed Effects). Z statistic images were thresholded using clusters determined by Z>2.0 and a corrected cluster significance threshold of *p* = 0.05 (using Gaussian Random Field Theory).

Analyses for the entire group were performed by computing linear t-contrasts between selected experimental conditions for the lexical decision task for each participant, which were then entered into a random effects two-sample t-test. To examine the correlation between behavioral improvement and activation increase, as well as interaction effects between increases in the TG and WG, we ran second-level (fixed effects) analyses for each subject to calculate the differences between activation patterns (pre vs. post activation). Subsequently, group level analyses (mixed effects) were run, including the number of incorrect responses inside the scanner as variable of no interest.

## Results

The main findings of this study are summarized in Figures, while the presented Tables S1 and S2 should provide additional information to the text and the Figures.

### 1. Baseline differences in brain activation patterns (Pre-Intervention)

**Figure 2:**
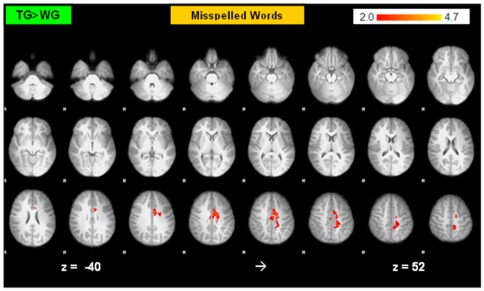
Baseline Comparison of the TG and WG during misspelled words. (Z>2.0; P corrected; P = 0.05). R = right.

To test our first hypothesis, we looked for group differences (TG, WG and CG) prior to the intervention (pre-test). Contrasts of the lexical decision tasks (1: correctly spelled words, 2: misspelled words, 3: pseudowords) versus fixation were computed. We analyzed whether the two groups with comparatively poor spelling and reading abilities (TG, WG) displayed comparable brain activation patterns before the intervention. In addition, we also investigated potential differences in brain activation between poor spellers and readers (TG, WG) and good spellers and readers (CG). The number of incorrect responses inside the scanner was included as variable of no interest. In the following section all significant differences of activation are reported:

The comparison of the two groups with comparatively poor spelling and reading abilities revealed increased activation in the left precuneus and left anterior cingulate gyrus for the TG compared to the WG during processing of misspelled words before the intervention (Figure 2; Table S1). No differences of activation between the TG and WG were found during the other conditions.

The comparison of the CG and the groups with comparatively poor spelling and reading abilities (TG and WG) revealed increased activation in left occipito-temporal regions and in the cerebellum for the CG during all three different lexical decision conditions (Figures S1, S2, S3). Beyond that, the CG exhibited increased activation in the left lateral occipital cortex, left inferior temporal gyrus, and left hippocampus relative to the TG (during processing pseudowords; Figure S2), and increased activation in the bilateral lateral occipital cortex and bilateral temporal regions compared to the WG (during processing of correctly spelled words and pseudowords; Table S1).

**Figure 3:**
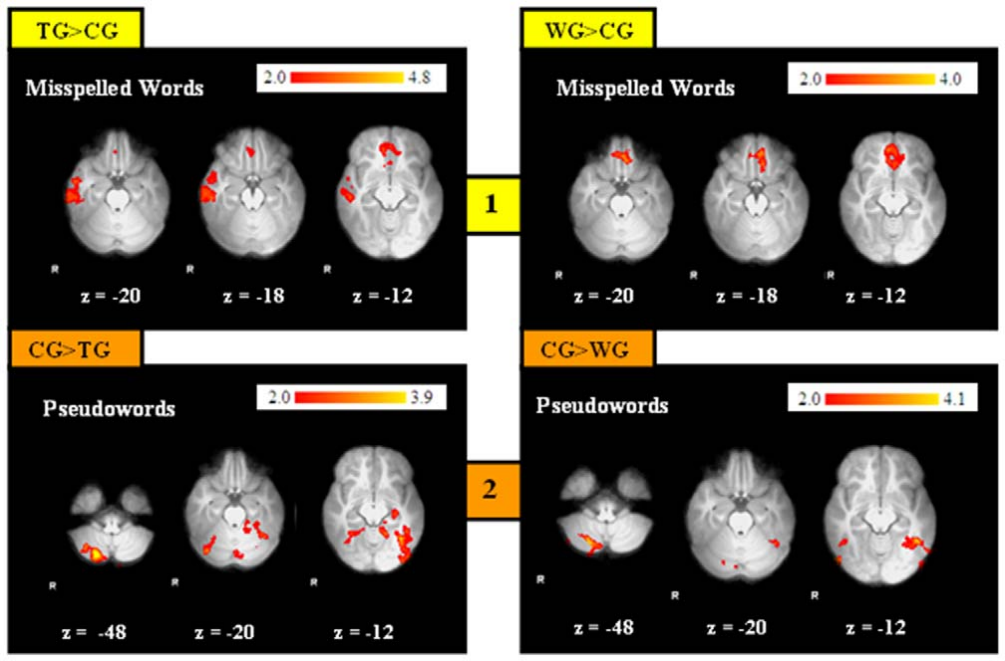
Baseline Comparison of Poor Spellers (Readers) vs. Controls. Pre-Intervention: 1: Activation during the condition misspelled words (relative to rest), 2: Activation during the condition pseudowords (relative to rest). Figures on the left represent contrasts between controls and the TG, and figures on the right contrasts between controls and the WG (Z>2.0; P corrected; P = 0.05). R = right. A more detailed representation of these contrasts is presented in Figure S2 and Figure S3.

Furthermore, increased activation during the processing of misspelled words for the groups with comparatively poor spelling and reading abilities (TG and WG) compared to the CG was observed in the precuneus, right posterior paracingulate gyrus and in the frontal medial gyrus. Beyond that the TG exhibited increased activation in right frontal areas and right temporal regions (Table S1, Figure 3 and S3).

### 2. Effects of the Intervention

#### 2.1. Behavioral Results

**Figure 4:**
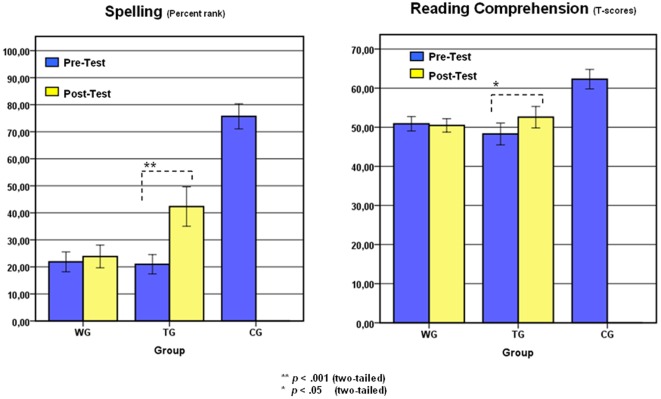
Behavioral Effects of the Training. Spelling (percentile rankings of the HSP) and reading comprehension (ELFE T-scores). For descriptive reasons, the pre-test scores of the CG group are presented.

To investigate the behavioral effects of the intervention, we computed a 2×2 ANOVA for repeated measures on the HSP spelling scores in considering TIME (pre- and post-test) as within subjects variable and GROUP (TG and WG) as between subjects variable. We observed a significant interaction between TIME and GROUP (*F*_(1,18)_ = 15.42; *p*<.001; *η_p_^2^* = .46), revealing increases in spelling performance only for the TG (Figure 4). With respect to reading, a 2×2 ANOVA for repeated measures on the SLS reading scores (indicative of reading speed) revealed a significant main effect of TIME (*F*_(1,18)_ = 8.79; *p*<.05; *η_p_^2^* = .33) indicating generally higher scores in the post- than in the pre-test. No significant interaction involving experimental group emerged. For reading comprehension (ELFE), the ANOVA yielded a significant interaction between TIME and GROUP (*F*_(1,18)_ = 4.52; *p*<.05; *η_p_^2^* = .20), revealing performance increases only for the TG (Figure 3). An overview of descriptive statistics is presented in Table 1.

#### 2.2. Behavioral Performance during fMRI

In order to investigate task performance during fMRI (measured by response accuracy in the lexical decision tasks), a 2×2 ANOVA for repeated measures yielded a significant main effect of TIME (*F*_(1,18)_ = 6.89; *p*<.05; *η_p_^2^* = .28), indicating generally higher scores in the post- than in the pre-test. The TIME by GROUP interaction failed to reach statistical significance, although the mean values (reported in Table 2) suggested stronger increases in accuracy for the TG than for the WG. To investigate changes in reaction time (RT in seconds) a 2×2 ANOVA for repeated measures was computed. The TIME by GROUP interaction reached statistical significance (*F*_(1,18)_ = 6.4; *p*<.05; *η_p_^2^* = .26), indicating a stronger increase in RT for the WG (see Table 2).

**Table 2:** Performance during fMRI (correctly solved tasks as percentage and reaction time in seconds (RT).

Performance during fMRI				
Accuracy	TG	WG	CG	
Pre-Intervention	72.4 (+/−8.4)	70.7 (+/−10.0)	89.7 (+/−5.9)	**.000**
Post-Intervention	77.9 (+/−8.9)	73.5 (+/−12.6)	91.1 (+/−4.1)	**.001**
**RT**				
Pre-Intervention	1.4 (+/−0.2)	1.3 (+/−0.2)	1.3 (+/−0.2)	.282
Post-Intervention	1.6 (+/−0.3)	1.7 (+/−0.2)	1.3 (+/−0.3)	**.003**

Means and Standard Deviations (in brackets).

#### 2.3. Functional MRI Results

**Figure 5:**
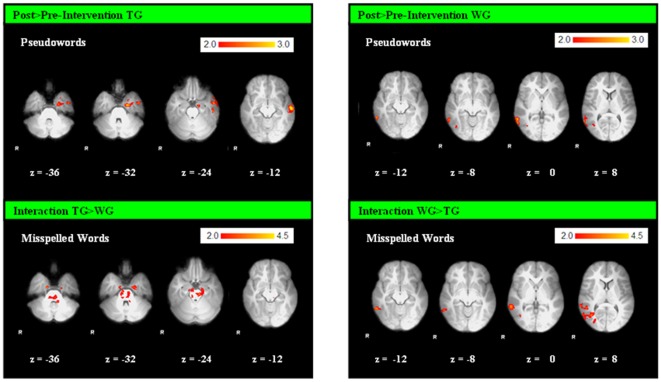
Changes of Activation in Poor Spellers. 1: Increases of activation after the intervention for the TG (left), compared to increases of activation without intervention for the WG (right), during the condition pseudowords. 2: Interaction Effect: Increased activation for the TG (compared to the WG) and for the WG (compared to the TG) during the condition misspelled words. (Z>2.0; P corrected; P = 0.05). R = right.

To test for changes in brain activation patterns post- compared to pre-intervention, we computed within group analyses for each group separately. Subsequently, to assess the training effects more specifically, we compared increases in activation (post>pre) for the TG, WG and CG during all lexical decision tasks. The within group comparison revealed increased activation in the precuneus for *all three* groups. Beyond that, for the *TG* increased activation in the right posterior cingulate, left inferior and middle temporal gyrus and left hippocampus and parahippocampal region related intervention was found (during processing pseudowords). For the *WG*, increases in the right lateral occipital cortex and right middle temporal cortex were observed (during all three conditions; Table S2, Figure 5). In the *CG* additional increases of activation in bilateral middle temporal and occipito-temporal regions at the second scan were found (during processing misspelled and pseudowords).

To investigate the effects of the intervention with respect to potential change of brain activation patterns, we compared increases in activation (post>pre) for the TG and WG. We observed a significant interaction effect, revealing increases in activation for the TG in the bilateral parahippocampal area and in the cerebellum (extending into the brain stem) during processing misspelled words, and increased activation for the WG in the precuneus, cerebellum, left frontal pole and right lateral occipital cortex and right parieto-temporal region (Table S2, Figure 5) during processing correctly spelled and misspelled words.

To assess the relation between improvement of spelling ability and increases in brain activation patterns, we computed whole-brain correlation analyses. We found negative correlations between improvement of spelling ability and activation increase in the cerebellum and right lateral occipital cortex, right lingual gyrus and right middle temporal gyrus in the TG during all three conditions (Table 3).

**Table 3:** Correlation: Increase of activation×less behavioral improvement in the TG.

CORRELATION: Increase of activation×less behavioral improvement in the TG					
	k	Z	x	y	z
**Correctly Spelled Words**					
R middle temporal gyrus	6805	3.4	58	−54	−10
R lateral occipital cortex	2467	3.27	32	−72	42
**Misspelled Words**					
R lateral occipital cortex	1808	3.08	30	−76	44
L cerebellum	1318	2.98	−40	−64	−28
**Pseudowords**					
L precentral gyrus	2782	3.31	−50	−10	40
R lingual gyrus	1665	2.83	14	−84	−10
R cerebellum	1381	3.03	22	−68	−22

Coordinates (in MNI standard space) and Activation Significance (Z statistics) of Local Maxima of Clusters, Z>2.0, P corrected P = 0.05.

## Discussion

This is the first study to investigate the effects of a morpheme-based spelling intervention on patterns of brain activity in children with comparatively poor spelling reading abilities using repeated fMRI. Behavioral improvements in spelling and reading comprehension were observed in the TG. Furthermore, increased activation in left temporal, parahippocampal and hippocampal regions after five weeks of intervention were noted in the TG. We interpret these changes as related to the recollection of the new learnt morpheme-based strategy; given the hippocampus and parahippocampal gyrus have relevance for memory recollection [44], [45]. In line with this notion, Krafnick et al. [46] recently reported increases in gray matter volume in the hippocampus in dyslexic children after an eight week reading intervention. The activation increases in the left inferior and middle temporal gyri could be indicative of enhanced reliance on concept retrieval, semantic processing and integration processes in the TG [47].

Conversely to *left* temporal and parahippocampal activation increases in the TG, the WG showed increases in *right* posterior regions (i.e. lateral occipital cortex, gyrus angularis, gyrus supramarginalis). Though speculative and preliminary, a possible explanation for the observed increases of activation in right posterior regions in the WG could be that they hint at (probably inefficient) compensatory cognitive mechanisms. It was found that dyslexic subjects (who represent a sample with most severe deficits in reading and spelling) showed increased activation in right posterior region [7], [11], presumably reflecting a serial grapheme-phoneme decoding compensation strategy [48]. Moll and Landerl [48] report that children with poor spelling and good reading abilities (similar to our samples) named pseudohomophones as quickly as their corresponding words, and their phonological awareness skills were adequate, suggesting that good reading ability in poor spellers might be based on highly efficient grapheme-phoneme decoding procedures. Due to the asymmetry in German language (grapheme-phoneme correspondence is high, but phoneme-grapheme correspondence is low) this strategy is not helpful for spelling difficulties, as different spellings for words with the same pronunciation (e.g. Wal/whale – Wahl/election) exist.

This interpretation is also strengthened by the observed negative correlations between behavioral improvement and activation increase in the cerebellum and right occipital and temporal regions in the TG. It seems that increased activation in the right posterior hemisphere correlates with less improvement of spelling ability due to intervention, which would further support the notion that reliance on the right posterior regions is probably related to inefficient compensation of poor spelling abilities.

The interpretation of these changes has to be done carefully, as the two groups of comparatively poor spellers and readers showed differences in activation patterns prior to the intervention in the precuneus and anterior cingulate. The precuneus is known to be part of the default-mode network [49]. Decreased activation in this region is related to attentive task engagement [49], reflecting rather general processing. Similarly, the anterior cingulate cortex is known as an important component of a neural network responsible for attention [50]. We therefore assume that the observed differences (TG vs. WG) at baseline are rather associated with differences in attentive task engagement than with differences in reading and spelling processes.

Remarkably, both groups of comparatively poor spellers and readers responded more slowly in the MRI session after the intervention, probably indicating that the TG and WG spent more time actually processing stimuli than the CG. However, on a purely descriptive level, there was a weak tendency towards a stronger improvement of accuracy of responses inside the scanner only for the TG (5.5%) as compared to the WG (2.8%) and CG (1.4%).

Prior to the intervention, both groups of comparatively poor spellers and readers showed increased activation in the precuneus and frontal medial cortex and relatively decreased activation of left occipito-temporal and cerebellar regions during a lexical decision task (relative to controls). Increased activation in the frontal medial region and paracingulate gyrus might be explained by a more effortful and attentionally guided reading strategy [15], [51] used by the TG and WG. Increased activation of the precuneus in children with spelling and reading impairments compared to non-impaired controls has also been found by others [7], [9]–[11], [17]. In non-impaired individuals the precuneus has been associated with attention, semantic processing and most notably with the default-mode network [47], [49], [52]. This region is active during conscious rest and deactivated during attentive task engagement. The general increase of activation in the precuneus found in all groups at the second scan thus suggests a general decrease in attention or excitement [47], [49], [52].

We also observed decreased activation of left occipito-temporal and cerebellar regions in comparatively poor spellers and readers relative to controls prior to the intervention. The left occipito-temporal region has been related to automatic and fluent reading [7], [9] and decreased activation has been found in multiple studies investigating dyslexia or reading impaired individuals. Several structural and functional imaging studies suggest cerebellar disruptions in individuals with dyslexia [53], [54], [55]. These have been related to semantic and phonological processing [56], skill automatization and learning [57]–[59] and linguistic performance [60], [61].

Some limitations of this study also have to be considered, when interpreting our results. First, the interpretation of the interaction effect (comparing within group changes of TG *and* WG) has to be done carefully, as these two groups showed differences in activation patterns prior to the intervention. However, the comparison of activation patterns post- vs. pre-intervention for each group separately revealed increased activation in parahippocampal regions for the TG, which was also observed by the interaction analyses. Second, a sample size of ten children per group might be regarded as rather small. While this may certainly compromise statistical power, it needs to be recognized that the employed study design (requiring children to participate in the training and to take part in several behavioral and fMRI testing sessions) imposed great efforts both on participants and the resources involved, together making studies of this kind difficult and rare. Third, a follow-up assessment including fMRI several months after the intervention would have been desirable to assess potential long-term effects of training outcome. Furthermore, the application of a more elaborated research design, such as the inclusion of a control activity (e.g. reading intervention), to investigate specific intervention effects would substantially improve these kinds of studies. In addition, future studies should investigate the impact of specific cognitive strategies applied by poor spellers and good spellers during a lexical decision task. It might possibly be the case that in our study comparatively poor spellers and readers relied on visual familiarity with real words, while good spellers actually attempted to detect misspelled real words.

Nonetheless, our study provides insights into the functional correlates of poor spelling ability and preliminary evidence for training-induced changes in brain function. We hope this work encourages future investigations into this area that also seek to overcome some of these shortcomings.

## Supporting Information

Figure S1 **Detailed overview of all activation differences during processing of correctly spelled words.** (Z>2.0; P corrected; P = 0.05). R = right.
(TIF)

Figure S2 **Detailed overview of all activation differences during processing of pseudowords.** (Z>2.0; P corrected; P = 0.05). R = right.
(TIF)

Figure S3 **Detailed overview of all activation differences during processing of misspelled words.** (Z>2.0; P corrected; P = 0.05). R = right.
(TIF)

Table S1 **fMRI Results Pre-Intervention.** Coordinates (in MNI standard space) and Activation Significance (Z statistics) of Local Maxima of Clusters, Z>2.0, P corrected P = 0.05. Comparison between the two spelling impaired groups (TG and WG), training group and controls (TG and CG) and waiting group and controls (WG and CG).
(DOC)

Table S2 **Changes of Activation related to Intervention (Within Group Comparison).** Post-Intervention vs. Pre-Intervention for the training group (TG) and waiting group (WG). Interaction effects of increases of activation (post-pre for TG vs WG). Coordinates (in MNI standard space) and Activation Significance (Z statistics) of Local Maxima of Clusters, Z>2.0, P corrected P = 0.05.
(DOC)
