# Supplementary figures and images for: Differences in Brain Function and Changes with Intervention in Children with Poor Spelling and Reading Abilities
Source: PLoS One. 2012 May 31;7(5):e38201. doi: 10.1371/journal.pone.0038201 (PMC3364962; doi:10.1371/journal.pone.0038201)

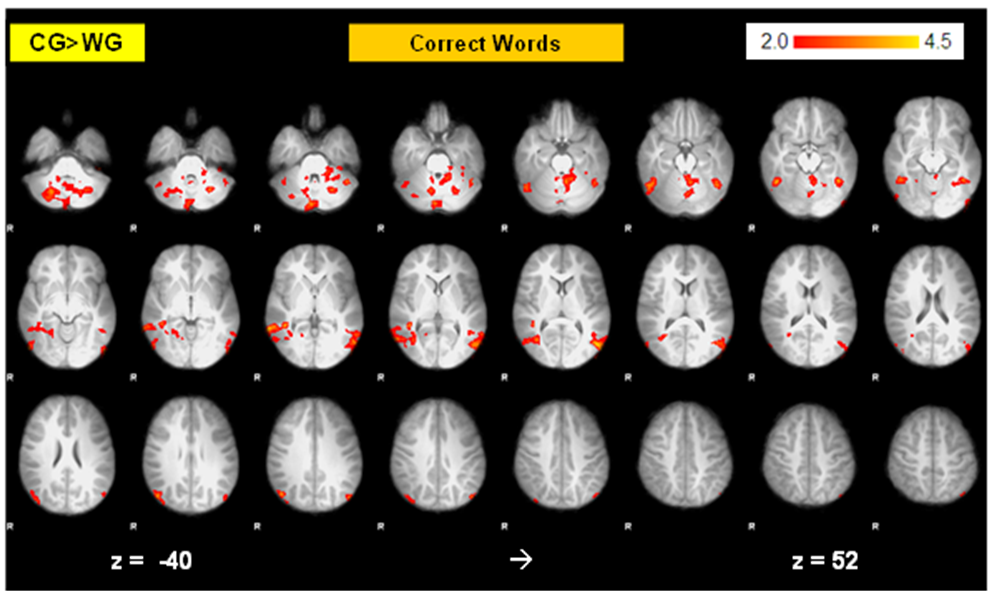

Supplement: Figure S1 — Detailed overview of all activation differences during processing of correctly spelled words. (Z>2.0; P corrected; P = 0.05). R = right. (TIF) [file pone.0038201.s001.tif]

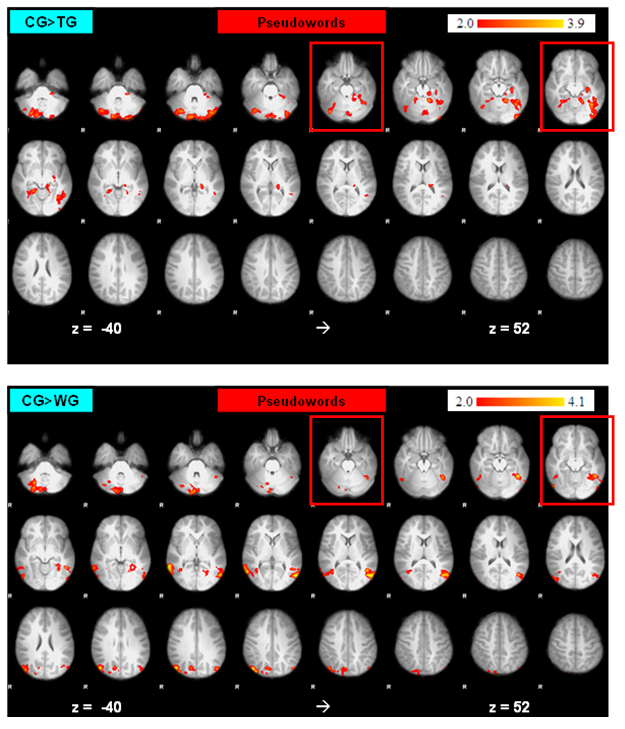

Supplement: Figure S2 — Detailed overview of all activation differences during processing of pseudowords. (Z>2.0; P corrected; P = 0.05). R = right. (TIF) [file pone.0038201.s002.tif]

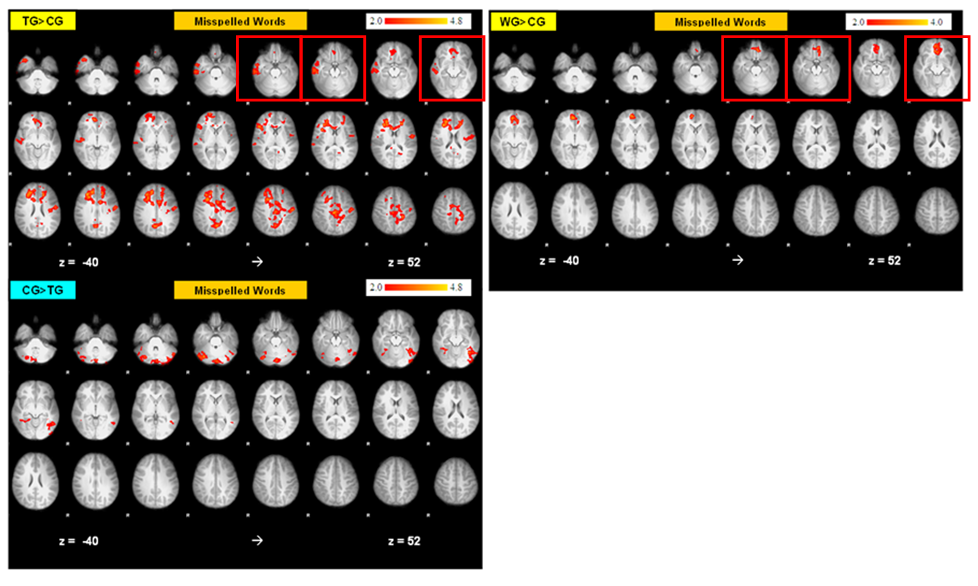

Supplement: Figure S3 — Detailed overview of all activation differences during processing of misspelled words. (Z>2.0; P corrected; P = 0.05). R = right. (TIF) [file pone.0038201.s003.tif]
