# Supplementary material for: Differences in Brain Function and Changes with Intervention in Children with Poor Spelling and Reading Abilities
Source: PLoS One. 2012 May 31;7(5):e38201. doi: 10.1371/journal.pone.0038201 (PMC3364962; doi:10.1371/journal.pone.0038201)
Supplement: Table S1 — fMRI Results Pre-Intervention. Coordinates (in MNI standard space) and Activation Significance (Z statistics) of Local Maxima of Clusters, Z>2.0, P corrected P = 0.05. Comparison between the two spelling impaired groups (TG and WG), training group and controls (TG and CG) and waiting group and controls (WG and CG). (DOC) [file pone.0038201.s004.doc]

| **Region (Local Maxima)** | **k** | **Z** | **x** | **y** | **z** |
| --- | --- | --- | --- | --- | --- |
| **Comparison between two spelling impaired groups** | | | | | |
| ***Correctly Spelled Words*** | ***-*** |  |  |  |  |
| **Misspelled Words** |  |  |  |  |  |
| **TG>WG** |  |  |  |  |  |
| Left precuneus | 1262 | 2.97 | -8 | -46 | 52 |
| Left anterior cingulate gyrus |  | 2.71 | -2 | -14 | 40 |
| ***Pseudowords*** | ***-*** |  |  |  |  |
| **Increased activation for controls compared to the spelling impaired groups** | | | | | |
| **Correctly Spelled Words** | | | | | |
| ***CG>TG*** | ***-*** |  |  |  |  |
| **CG>WG** |  |  |  |  |  |
| R cerebellum | 3177 | 4.01 | 22 | -72 | -48 |
| L cerebellum |  | 3.14 | -6 | -52 | -22 |
| R inferior temporal gyrus | 2019 | 3.67 | 48 | -52 | -16 |
| R middle temporal gyrus |  | 3.6 | 68 | -48 | 0 |
| R lateral occipital cortex |  | 3.38 | 50 | -74 | 30 |
| L lateral occipital cortex | 1643 | 3.77 | -52 | -72 | 8 |
| L inferior temporal gyrus |  | 3.57 | -44 | -50 | -14 |
| L occipito-temporal gyrus |  | 3.27 | -46 | -56 | -22 |
| **Misspelled Words** | | | | | |
| **CG>TG** |  |  |  |  |  |
| R cerebellum | 3744 | 4.21 | 24 | -76 | -48 |
| L occipito-temporal fusiform gyrus |  | 3.67 | -38 | -58 | -12 |
| ***CG>WG*** | ***-*** |  |  |  |  |
| **Pseudowords** | | | | | |
| **CG>TG** |  |  |  |  |  |
| R cerebellum | 3248 | 3.88 | 24 | -76 | -48 |
| L cerebellum |  | 3.62 | -38 | -78 | -30 |
| L occipito-temporal fusiform gyrus | 2122 | 3.79 | -38 | -58 | -12 |
| L inferior temporal gyrus |  | 3.35 | -44 | -50 | -16 |
| L lateral occipital cortex |  | 3.24 | -40 | -88 | -18 |
| L thalamus, L hippocampus |  | 2.96 | -16 | -36 | 2 |
| ***CG>WG*** | ***-*** |  |  |  |  |
| **Pseudowords** | | | | | |
| ***CG>TG*** | ***-*** |  |  |  |  |
| **CG>WG** |  |  |  |  |  |
| R lateral occipital cortex | 2664 | 4.02 | 48 | -76 | 34 |
| R middle temporal gyrus |  | 3.91 | 66 | -48 | 0 |
| L lateral occipital cortex | 2094 | 3.98 | -56 | -72 | 4 |
| L inferior temporal gyrus |  | 3.91 | -44 | -50 | -14 |
| L middle temporal gyrus |  | 3.42 | -46 | -58 | 8 |
| **Increased activation for the spelling impaired groups compared to controls** | | | | | |
| ***Correctly Spelled Words*** | ***-*** |  |  |  |  |
| **Misspelled Words** | | | | | |
| **TG>CG** |  |  |  |  |  |
| R cingulate gyrus (posterior) | 12279 | 4 | 4 | -22 | 44 |
| R frontal pole, R middle frontal gyrus |  | 3.96 | 32 | 36 | 28 |
| R middle temporal gyrus | 2184 | 3.27 | 64 | -18 | -20 |
| R temporal pole |  | 3.25 | 42 | 16 | -38 |
| R superior temporal gyrus |  | 3.24 | 48 | -16 | -6 |
| R parietal operculum |  | 3.21 | 56 | -24 | 20 |
| **Misspelled Words** | | | | | |
| **WG>CG** |  |  |  |  |  |
| R paracingulate gyrus (posterior) | 1517 | 3.37 | 4 | 48 | -4 |
| R frontal medial cortex |  | 3.23 | 8 | 48 | -10 |
| L frontal medial cortex |  | 3.13 | -4 | 42 | -22 |
| ***Pseudowords*** | ***-*** |  |  |  |  |

**TG = Training Group, WG = Waiting Group, CG = Control Group; k = number of voxels; R = right; L = left**
