# Supplementary material for: Differences in Brain Function and Changes with Intervention in Children with Poor Spelling and Reading Abilities
Source: PLoS One. 2012 May 31;7(5):e38201. doi: 10.1371/journal.pone.0038201 (PMC3364962; doi:10.1371/journal.pone.0038201)
Supplement: Table S2 — Changes of Activation related to Intervention (Within Group Comparison). Post-Intervention vs. Pre-Intervention for the training group (TG) and waiting group (WG). Interaction effects of increases of activation (post-pre for TG vs WG). Coordinates (in MNI standard space) and Activation Significance (Z statistics) of Local Maxima of Clusters, Z>2.0, P corrected P = 0.05. (DOC) [file pone.0038201.s005.doc]

| **Region (Local Maxima)** | **k** | **Z** | **x** | **y** | **z** |
| --- | --- | --- | --- | --- | --- |
| **WITHIN GROUP PRE-POST COMPARISON** | | | | | |
| **Training Group** | | | | | |
| ***Correctly Spelled Words*** | ***-*** |  |  |  |  |
| ***Misspelled Words*** | ***-*** |  |  |  |  |
| **Pseudowords** |  |  |  |  |  |
| **Posttest>Pretest** |  |  |  |  |  |
| L middle temporal gyrus | 1483 | 3.09 | -60 | -14 | -12 |
| L inferior temporal gyrus |  | 2.85 | -54 | -24 | -22 |
| L parahippocampal |  | 2.79 | -22 | -6 | -32 |
| R precuneus | 1309 | 2.9 | 12 | -58 | 10 |
| L precuneus |  | 2.7 | 0 | -68 | 20 |
| R cuneal cortex |  | 2.63 | 6 | -70 | 20 |
| R posterior cingulate gyrus |  | 2.61 | 2 | -52 | 22 |
| **Waiting Group** | | | | | |
| **Correctly Spelled Words** | | | | | |
| **Posttest>Pretest** |  |  |  |  |  |
| R precuneus | 2580 | 3.14 | 8 | -74 | 40 |
| L precuneus |  | 3.02 | -2 | -54 | 48 |
| **Misspelled Words** |  |  |  |  |  |
| **Posttest>Pretest** |  |  |  |  |  |
| R lateral occipital cortex | 1817 | 2.89 | 36 | -82 | 38 |
| L precuneus | 1262 | 3.11 | -2 | -74 | 46 |
| R precuneus |  | 2.95 | 4 | -58 | 40 |
| **Pseudowords** |  |  |  |  |  |
| **Posttest>Pretest** |  |  |  |  |  |
| R precuneus | 1749 | 3.1 | 4 | -56 | 46 |
| L precuneus |  | 3.05 | -2 | -58 | 48 |
| R lateral occipital cortex | 1186 | 2.91 | 50 | -78 | 30 |
| R middle temporal cortex |  | 2.81 | 66 | -50 | -2 |
| **INTERACTION: INCREASE OF ACTIVATION (TG vs WG)** | | | | | |
| **Increased activation for the TG** | | | | | |
| ***Correctly Spelled Words*** | ***-*** |  |  |  |  |
| **Misspelled Words** |  |  |  |  |  |
| Brain stem | 1566 | 3.44 | -4 | -36 | -46 |
| R parahippocampal cortex |  | 3.38 | 18 | -8 | -38 |
| ***Pseudowords*** | ***-*** |  |  |  |  |
| **Increased activation for the WG** | | | | | |
| **Correctly Spelled Words** |  |  |  |  |  |
| L precuneus | 6326 | 4.67 | -2 | -74 | 48 |
| R precuneus |  | 4.07 | 2 | -80 | 44 |
| R cerebellum | 1778 | 3.95 | 12 | -76 | -44 |
| L cerebellum |  | 3.75 | -12 | -80 | -42 |
| L frontal pole | 1246 | 3.66 | -36 | 60 | 8 |
| **Misspelled Words** |  |  |  |  |  |
| R parieto-temporal | 2727 | 4.5 | 52 | -36 | 36 |
| gyrus angularis; gyrus supramarginalis |  | 3.7 | 44 | -44 | 24 |
| R lateral occipital cortex |  | 3.69 | 36 | -78 | 38 |
| R middle temporal gyrus |  | 3.64 | 60 | -50 | -10 |
| ***Pseudowords*** | ***-*** |  |  |  |  |

**TG = Training Group, WG = Waiting Group; k = number of voxels; R = right; L = left**
